# Swim Training Attenuates Inflammation and Improves Insulin Sensitivity in Mice Fed with a High-Fat Diet

**DOI:** 10.1155/2017/5940732

**Published:** 2017-09-10

**Authors:** Guangzeng Zhang, Pengfei Yu, Xiaomeng Liu

**Affiliations:** Institute of Neuroscience and Translational Medicine, College of Life Sciences and Agronomy, Zhoukou Normal University, Zhoukou, Henan 466001, China

## Abstract

Exercise could afford multiple beneficial effects on obesity-related metabolic disorders. To address this issue, C57BL/6J mice were used to investigate the effects of 13 weeks of swim training on HFD-induced obesity and related insulin resistance and inflammation. Our results show that swim training can significantly prevent HFD-induced weight gain and increase resting energy expenditure without affecting food intake. The insulin sensitivity was enhanced in the HFD + swim group than in the HFD + sedentary group. Moreover, swim training considerably decreased serum LPS content and downregulates epididymis white adipose tissue (eWAT) expression of the inflammatory mediator *Tnf-α*, *Il-6*, and *Mcp-1*. In summary, 13 weeks of swim training could reverse HFD-induced metabolic disorders including insulin resistance and inflammation.

## 1. Introduction

Obesity has become a prevalent disease; type II diabetes and related complications are directly related to it [1]. Obesity can usually cause insulin resistance, which subsequently contribute to the development of a variety of complications such as type 2 diabetes, hypertension, dyslipidemia, cardiovascular disease, and cancers [2, 3]. Therefore, high mortality caused by obesity and its complications have been recognized. For example, in 2010, up to 3.4 million of deaths worldwide were closely linked to obesity [4]. These data suggest that obesity has become one of the most important public health problems.

Mounting evidence indicates that obesity is highly associated with low-grade, chronic systemic inflammation [5–7]. Several hypotheses have been proposed to link obesity and inflammation. One of them is that, as white adipose tissue (WAT) expanded during the progression from lean to obese state, microhypoxia can occur; the hypoxia environment causes adipocyte stress and death which further trigger macrophage recruitment and inflammation [8, 9]. Besides, inflammation also causes obesity. After excessive HFD exposure, the gut microbiota pattern may be changed and released lipopolysaccharide (LPS) into the circulation which further contributes to systematic inflammatory signals and body weight gain [10–12]. During these obesity-related inflammatory responses, several inflammatory mediators such as tumor necrosis factor-*α* (TNF-*α*), monocyte chemoattractant protein-1 (MCP-1), and interleukin-6 (IL-6) are generally involved [13–17]. A number of studies have showed that these inflammatory mediators are the crucial factors triggering the obesity-related complications aforementioned. For instance, TNF-*α*-treated adipocytes exhibit impair insulin signaling and subsequently decreased glucose uptake [18–20]. Thus, lowering obesity-related inflammation is regarded effective to combat obesity and related complications.

A widely accepted consensus is that lifestyle changes are fundamentally effective to combat obesity and related metabolic syndrome [21, 22]. Among these, the physical activity, with minor side effects, is regarded as a natural strong anti-inflammatory and metabolism-improving strategy [23]. However, whether the swim training exerts these beneficial effects on diet-induced obesity remains largely unknown. The present study was designed to investigate the effect of long-term swim training on obesity and obesity-related inflammation and insulin resistance in mice fed with a high-fat diet.

## 2. Materials and Methods

### 2.1. Animals and Diets

Principles of laboratory animal care were followed, and all procedures were conducted according to the guidelines established by the National Institutes of Health, and every effort was made to minimize suffering. This study was approved by the Animal Ethics Committee of Zhoukou Normal University, Zhoukou. Male C57BL/6J mice were purchased from the Vital River Laboratory Animal Technology Co Ltd. (Beijing, China) at 4 weeks of age. The mice in the group were allowed to adapt to each other for one week; 11 mice with similar body weights were selected and received a high-fat diet (HFD, 60% of calories derived from fat, Research Diets, New Brunswick, NJ; D12492). Ten weeks later, the mice were divided into sedentary group (HFD, *n* = 6) and swim training group (HFD + swim, *n* = 5). The atmosphere environment was controlled at 22 ± 2°C and 55 ± 10% relative humidity with a 12 h light/dark cycle. All mice were given free access to diet and water.

### 2.2. Exercise Protocols

Swim training was conducted for 13 weeks in a plastic cylindrical pool of 45 cm in diameter and 60 cm of deep, with a water temperature of 35 ± 2°C. During the initial training period, the duration of daily training was increased from 10 to 60 min. Then, the mice underwent exercise regularly for 60 min/day, 5 days/week. The mice were killed in the last 24 hours after swimming.

### 2.3. Measures of Body Weight, Blood Glucose, Food Intake, and Energy Expenditure

Body weight was measured weekly during the exercise period. Food intake was measured for seven consecutive days after two days of acclimation, and the data was present as the energy expenditure in one day. Oxygen consumption was measured for two consecutive days using an animal oxygen consumption records (IntelliCage, America Thermo Scientific).

### 2.4. Glucose Tolerance Test (GTT) and Insulin Tolerance Test (ITT)

Glucose and insulin tolerance tests were performed at the end of experiment. Basal blood glucose levels (0 min) before the injection of glucose or insulin were measured after 16 hours (GTT) or 6 hours (ITT) fasting. After the animals were intraperitoneally injected with either 1.5 g/kg glucose or 1 U/kg insulin, the blood samples were collected from the tail vein for measurements of the blood glucose levels at 30, 60, 90, and 120 min and 15, 30, 45, and 60 min, respectively.

### 2.5. Serum Preparation and LPS Content Measurement

Mice were anesthetized with sodium pentobarbital and sacrificed by cervical dislocation. The serum of the blood sample was isolated by centrifugation at 12000 rpm for 15 min at 4°C. Serum LPS content was measured using commercial kits (Yansheng Biochemistry Co. Ltd., Shanghai, China), according to the manufacturer's instructions.

### 2.6. Extraction of RNA

The eWAT in liquid nitrogen will be broken and placed in the centrifuge tube. After adding TransZol Up (trans), we mix the chloroform and centrifuge the pipe. The supernatant was transferred to a new centrifuge tube. The pipe added isopropyl alcohol and washed with 75% ethanol; then, the RNA dissolved with RNA solution.

### 2.7. RT-qPCR

Total RNA from epididymis WAT (eWAT) was extracted using a Trizol reagent (Transgen, Beijing, China). Complementary DNA (cDNA) was synthesized using SuperScript II Reverse Transcriptase, random primers, and dNTP. Real-time PCR was carried out using the CFX Connect Real-Time PCR Detection System (Bio-Rad).

Relative differences between groups were calculated based on the equation relative quantification = 2 − ^△△^Ct [24]. The amplification efficiencies of the gene of interest and the housekeeping gene were equivalent, and the expression of GAPDH did not change between groups. The following primers in RT-qPCR are used: GAPDH, 5′-AGGTCGGTGTGAACGGATTTG-3′ (forward), 5′-TGTAGACCATGTAGTTGAGGTCA-3′ (reverse); *Tnf-α*, 5′-TGGGCCTCATGCACCACC-3′ (forward), 5′-GAGGCAACCTGACCACTCTCCCT-3′ (reverse); *Mcp-1*, 5′-AGAGAGCCAGACGGGAGG-3′ (forward), 5′-CAGCAGGCCCAGAAGCAT-3′ (reverse); *Il-6*, 5′-AGACAAAGCCAGAGTCCTTCAGAGA-3′ (forward), 5′-GCCACTCCTTCTGTGACTCCAGC-3′ (reverse).

### 2.8. Statistics

All data were presented as mean ± SEM. For all comparisons, unpaired two-tailed tests were performed using GraphPad Prism Version 5. *P* values less than 0.05 were considered to be statistically significant.

## 3. Results

### 3.1. Swim Training Improves Basal Metabolic Parameters

**Figure 1:**
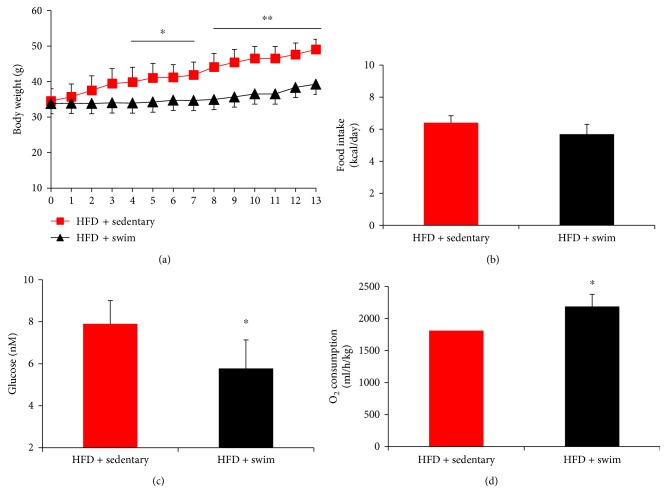
Swim training improves basal metabolic parameters. Swim training prevented HFD-induced body weight gain (a) and decreased blood glucose level (c); the energy expenditure in term of O_2_ consumption (b) was enhanced in the context of food intake (d) not changed significantly in HFD + swim group than in HFD + sedentary group. Data are shown as mean ± SEM (*n* = 4–6/group). ^∗^*P* < 0.05 and ^∗∗^*P* < 0.01.

Swim training alleviated HFD-induced body weight gain (Figure 1(a)). This significant difference (*P* < 0.05) between the HFD + swim and HFD + sedentary groups can even be found from the fourth training week. After 13 weeks of training, the average body weights of the two groups were 49.05 ± 2.9 g and 39.26 ± 4.1 g, showing a considerably significant difference. Besides, swim training remarkably alleviates the high blood glucose level induced by HFD (Figure 1(c)). To investigate whether swim training increased energy expenditure, the food intake and O_2_ consumption were measured. As shown in Figures 1(b) and 1(d), compared with the HFD + sedentary group, the HFD + swim group showed an elevated O_2_ consumption with the food intake unchanged.

### 3.2. Swim Training Improves Glucose Homeostasis

**Figure 2:**
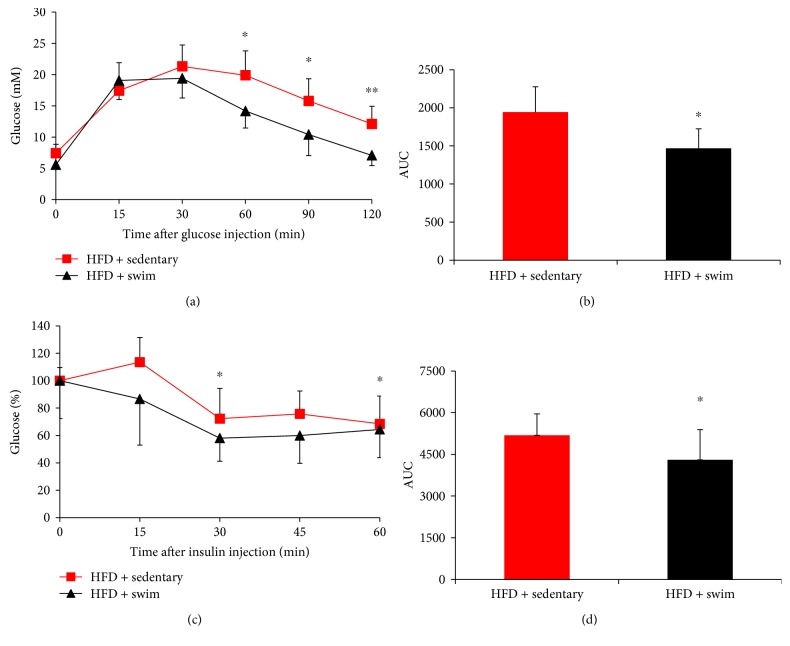
Swim training improves glucose homeostasis. Glucose homeostasis was significantly improved in mice after swim training, as evidenced by GTT (a), the AUC of GTT (b), ITT (c), and the AUC of ITT (d). Data are shown as mean ± SEM (*n* = 4–6/group). ^∗^*P* < 0.05 and ^∗∗^*P* < 0.01.

The suppression of body weight gain and enhanced energy expenditure in the HFD + swim group suggests that swim training might affect whole-body insulin sensitivity. To test this, GTT and ITT were performed. Results showed that swim training significantly improved glucose homeostasis (Figure 2). In detail, the area under the curve (AUC) value of GTT was significantly decreased in HFD + swim group than in HFD + sedentary group (*P* < 0.05, Figure 2(b)); in parallel, the AUC of ITT also showed significant improvement of insulin sensitivity in HFD + swim group (*P* < 0.05, Figure 2(d)).

### 3.3. Swim Training Decreases Serum LPS Content and Downregulates Expressions of Inflammatory Mediators in eWAT

**Figure 3:**
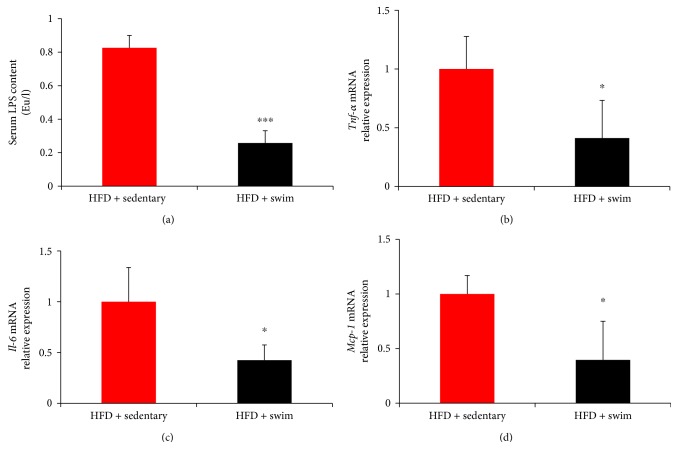
Swim training decreases serum LPS content and downregulates expressions of inflammatory mediators in eWAT. Swim training significantly decreased serum LPS content (a) and suppressed the expressions of *Tnf-α* (b), *Il-6* (c), and *Mcp-1* (d). Data are shown as mean ± SEM (*n* = 3–6/group). ^∗^*P* < 0.05 and ^∗∗∗^*P* < 0.001.

LPS plays a crucial role in diet-induced obesity and insulin resistance. To investigate the effect of swim training on LPS content, the serum LPS content in mice was measured. Our results showed that, compared with HFD + sedentary group, the HFD + swim group has considerably decreased serum LPS content (*P* < 0.001, Figure 3(a)). In addition, the expression levels of inflammatory mediators *Tnf-α*, *Mcp-1*, and *Il-6* in eWAT were measured. Our results showed that swim training significantly suppressed the expressions of all the three genes (*P* < 0.05, Figures 3(b)–3(d)).

## 4. Discussion

Moderate exercise could afford multiple beneficial effects on metabolic disease from obesity. In the present study, we clarified that swim training could effectively increase energy expenditure and prevent body weight gain in HFD-treated mice. Moreover, systemic inflammation and insulin resistance were also alleviated in exercise group compared to those in the sedentary group.

Weight loss is one effective strategy to combat obesity and related complications. Dietary modification, comprehensive lifestyle change including diet and exercise, and bariatric surgery are all recommended to reach weight loss [22, 25]. Among them, the exercise, with minor side effects, is concerned by the public [23]. During the course of weight loss by exercise, whether the resting energy expenditure increases or not remains a debate in terms of mice. Several reports point that this energy expenditure is decreased (Leibel et al., 1995; Melanson, 2016). In contrast, Evangelista et al. [26] reported that the resting energy expenditure is increased. In the present study, compared with the sedentary group, the exercise group showed significantly elevated resting energy expenditure without affecting food intake.

LPS is metabolic endotoxin released by gut microbiota. After the LPS is produced and released into the circulation, inflammatory response often occurs through LPS/CD14 system and subsequently causes body weight gain and insulin resistance [10, 11]. Therefore, lowering serum LPS content is regarded as a potent strategy for the control of body weight and related metabolic diseases [10]. Previous studies reported that high-fat feeding can significantly increase the proportion of LPS-containing microbiota in the gut [10, 11]. Interestingly, treadmill exercise could reverse HFD-induced intestinal dysbacteriosis and decrease the proportion of LPS-containing microbiota [27]. These observations suggest that, in the present study, the weight loss and improvement of insulin resistance might be closely correlated with gut microbiota probably changed by swim training.

In addition to metabolic endotoxin, some other factors have been reported responsible for systematic inflammation in obese individuals. One of these is that, as the adipose tissue expanded in obese individuals, the microhypoxia can occur which triggers macrophage recruitment and upregulates the expression of proinflammatory such as *Tnf-α* [8, 16, 28, 29]. Given that the eWAT is closely associated with impaired peripheral insulin sensitivity and this tissue-specific insulin resistance might lead to the overall disease state in type 2 diabetes [30–32], we investigated the relationships between swim training and eWAT inflammatory gene expression in mice fed with a HFD. We observed that 13 weeks of swim training significantly reduced the expression of inflammatory mediators *Tnf-α* and *Il-6* in eWAT, as well as the expression of the immune marker *Mcp-1* which can promote macrophages recruitment to eWAT in HFD-treated mice. However, discrepancies exist between our results and the results in Baynard et al. [33] who found no differences in eWAT expression of the *Tnf-α* and *Mcp-1* between the exercised and sedentary mice. However, in Baynard et al. [33], only 6 weeks of treadmill training is conducted, suggesting that the exercise time may be a crucial factor for exercise to exert its beneficial effects on metabolic disorders.

In conclusion, swim training could effectively prevent HFD-induced weight gain by increasing resting energy expenditure and decreasing serum LPS content. Moreover, HFD-induced insulin resistance can also be reversed by long-term swim training.
